# COVID-19 pandemic impairs medical care of vasculitis patients in Germany: Results of a national patient survey

**DOI:** 10.3389/fmed.2022.1103694

**Published:** 2023-01-09

**Authors:** Anna Kernder, Tim Filla, Kirsten de Groot, Bernhard Hellmich, Julia Holle, Peter Lamprecht, Frank Moosig, Nikolas Ruffer, Christof Specker, Stefan Vordenbäumen, Matthias Schneider, Gamal Chehab

**Affiliations:** ^1^Department of Rheumatology, University Hospital Düsseldorf, Medical Faculty of Heinrich Heine University, Düsseldorf, Germany; ^2^Hiller Research Center, University Hospital Düsseldorf, Medical Faculty of Heinrich Heine University, Düsseldorf, Germany; ^3^Medical Clinic III, Sana Klinikum Offenbach GmbH, Offenbach, Germany; ^4^Klinik für Innere Medizin, Rheumatolgie und Immunologie, Medius Klinik Kirchheim, University Tübingen, Kirchheim unter Teck, Germany; ^5^Rheumazentrum Schleswig-Holstein Mitte, Neumünster, Germany; ^6^Department of Rheumatology and Clinical Immunology, University of Lübeck, Lübeck, Germany; ^7^Department of Rheumatology and Immunology, Klinikum Bad Bramstedt GmbH, Bad Bramstedt, Germany; ^8^III Department of Medicine, University Medical Center Hamburg-Eppendorf, Hamburg, Germany; ^9^Department of Rheumatology and Clinical Immunology, KEM Kliniken Essen-Mitte, Essen, Germany; ^10^Department of Rheumatology, St. Elisabeth-Hospital Meerbusch-Lank, Meerbusch, Germany

**Keywords:** systemic vasculitis, COVID-19, vaccination coverage, health care, patient survey

## Abstract

**Objective:**

To analyze the impact of the COVID-19 pandemic on medical care and vaccination acceptance of vasculitis patients in Germany.

**Methods:**

A web-based national survey was developed by rheumatology centers and vasculitis patient advocacy groups. The survey was distributed nationwide by mail and flyers and could be accessed via a QR-code or weblink from December 2021 to April 2022. Descriptive statistics [mean, median, standard derivation (SD), 25%, 75% quantile] were calculated. 95% confidence intervals were presented for responses that were directly related to the impact of COVID-19 on parameters associated with vasculitis patient care.

**Results:**

The online survey was completed by 117 patients with small and large vessel vasculitis [granulomatosis with polyangiitis (*n* = 69), eosinophilic granulomatosis with polyangiitis (*n* = 16), microscopic polyangiitis (*n* = 12), giant cell arteritis (*n* = 17) and Takayasu's arteritis (*n* = 3)]. Prescheduled rheumatological appointments had been canceled due to the COVID-19 pandemic in 12.6% of the respondents [95% confidence interval (CI), 7.3–20.0%); in 9% (95% CI, 4.5–15.6%)] appointments had been replaced by digital services. Therapeutic regimens were changed (shifted, reduced, or discontinued) due to the pandemic in 15.5% (95% CI 9.5–22.2%). Vaccination coverages were generally high compared to patients with other rheumatic diseases and the general population. Highest vaccination coverage was observed against COVID-19 (98.1% 95% CI 93.9–99.6%).

**Conclusion:**

Vasculitis patients experienced changes in medical care during COVID-19 pandemic such as cancelation of prescheduled rheumatology appointments and modifications in therapeutic regimens. The overall acceptance rate for vaccination was comparatively high, particularly for vaccination against COVID-19.

## Introduction

The care of vasculitis patients during the ongoing COVID-19 pandemic represents an extraordinary challenge: Once diagnosed, patients require regular follow-up visits to assess treatment responses, track symptoms, perform blood tests, monitor potential adverse events and comorbidities, and re-evaluate treatment indications (1).

At the beginning of the pandemic, risk factors associated with mortality and morbidity associated with COVID-19 infections were unknown and optimal management of immunosuppressive therapy was uncertain. This resulted in widespread anxiety and a high rate of self-isolation among patients diagnosed with rheumatic and musculoskeletal diseases (RMDs) (2, 3).

German (Deutsche Gesellschaft für Rheumatologie, DGRh) and European (European Alliance of Associations for Rheumatology, EULAR) organizations ultimately developed a set of recommendations and provided guidelines to address these concerns (4–7).

Specifically, these guidelines recommended to balance the benefits of regular visits (monitoring of disease activity, complications and comorbidities) and the risks associated with direct in-person consultations. Consequently, extended follow-up intervals could be considered for patients in a stable disease status (4).

Ultimately, vaccinations against COVID-19 were approved and recommended for patients diagnosed with RMDs (7–9). Reported vaccination rates in these patients ranged from 30.2 to 80% between June and October 2021 (10–12).

Against this background, our study aims to examine the impact of COVID-19 on the medical care of vasculitis patients across specialized centers in Germany and to evaluate their overall acceptance of COVID-19 vaccination.

## Methods

A web-based national survey was developed by rheumatology centers in collaboration with vasculitis patient advocacy groups. An expert panel identified two areas of interest to investigate: (1) medical care of vasculitis patients during the COVID-19 pandemic and (2) vaccine acceptance.

After performing a literature search, members of the panel designed a questionnaire based on the standard operating procedures outlined by the EULAR recommendation task force (13). The questionnaire was shared with members of vasculitis patient advocacy groups who reviewed the draft version and provided critical insight and patient perspectives.

The web-based survey (LimeSurvey, https://www.limesurvey.org/) was distributed nationwide by members of patient advocacy groups and clinical rheumatologists *via* flyers and mail. The survey was accessible *via* a QR code or weblink from December 9, 2021, to April 19, 2022. Informed consent was obtained from all participants prior to study participation. Patients with self-reported diagnoses of small and large vessel vasculitis, including granulomatosis with polyangiitis (GPA), eosinophilic granulomatosis with polyangiitis (EGPA), microscopic polyangiitis (MPA), giant cell arteritis (GCA) and Takayasu arteritis (TAK) were included in the analysis. Participation was voluntary and there were no incentives provided. Internet protocol (IP) addresses were used to identify potential duplicate entries. Responses from incomplete questionnaires were included in the analysis as appropriate. This resulted in differences of the overall number of patients for different questions within the questionnaire. The respective total numbers are indicated in the text.

The study methodology and results are reported according to the Checklist for Reporting Results of Internet E-Surveys (14).

### Statistical analysis

The data was collected anonymously. For data description, either absolute or relative numbers given by the percentage of all observations for binary covariates were used. For continuous covariates, the distribution for symmetry and potential outliers were visually checked. If both conditions were sufficiently met, covariate was calculated as mean (±SD), otherwise as median [25%; 75%quantile]. 95% confidence intervals were calculated using Jeffreys equal-tailed intervals as it showed improved performance in comparison with confidence intervals based on the normal approximation in settings with low prevalence (15). All missing data were assumed as missing at random.

Daily rates of COVID-19 prevalence in Germany were provided by the Robert-Koch-Institute (available at https://corona.rki.de) and used to evaluate findings on days corresponding to the time points the patients answered the questionnaire^1^. Average prevalence rates were compared with the prevalence rates determined for study participants.

To interpret the COVID-19 vaccination rates in our cohort, patients were asked about other recommended vaccinations. Recommended standard vaccinations (16) against tetanus and diphteria were used to compare vaccination status among participants. Influenza and pneumococcal vaccination are only recommended for selected patients in Germany. The frequencies presented were calculated based on the number of persons eligible for the respective vaccination according to the recommendations of the German Society for Rheumatology and the German Standing Committee on Vaccination. Our findings were compared to data obtained from the Association of Statutory Health Insurances from 2020 to 2021 presenting the vaccination coverage of persons eligible for the respective vaccination in the total population (Kassenärztliche Vereinigungen, RKI Vaccination Surveillance) (17).

All calculations were performed using the “R” software environment Version 4.1.2.

The study was approved by the Ethics Committee of the medical faculty of the University of Duesseldorf, Germany (2021–1405).

### Patient and public involvement

The research question and questionnaire was developed by rheumatology centers in collaboration with vasculitis patient advocacy groups who provided critical insight and patient perspectives. The web-based survey was distributed nationwide by members of patient advocacy groups and clinical rheumatologists *via* flyers and mail. Results will be disseminated *via* the patient advocacy groups using graphical presentation of the results (Supplementary material 1).

## Results

### Participant details

The online survey was answered by 116 patients, who were diagnosed with small and large vessel vasculitis (GPA, EGPA, MPA, GCA or TAK). GPA was the most prevalent disease (*n* = 69/117, 59.0%), followed by GCA (*n* = 17/117, 14.5%). The mean age of the study respondents was 56.3 (±15.6) years. The majority of study participants were female (*n* = 73/117, 62.4%). Additional demographics and patient characteristics are presented in Table 1.

**Table 1:** Characteristics of the patient cohort.

	**Overall**	**Antineutrophil cytoplasmic antibody (ANCA)-associated vasculitis**	**Large vessel vasculitis**
*n* (%)	**117 (100)**	**97 (82.9)**	**20 (17.1)**
Granulomatosis with polyangiitis (GPA)		69 (71.1)	
Eosinophilic granulomatosis with polyangiitis (EGPA)		16 (16.5)	
Microscopic polyangiitis (MPA)		12 (12.4)	
Giant cell arteritis (GCA)			17 (85.0)
Takayasu's arteritis			3 (15.0)
Age; mean (SD)	56.3 (15.6)	55.5 (15.4)	60.0 (16.3)
**Sex;** ***n*** **(%)**			
Female	73 (62.4)	56 (57.7)	17 (85.0)
Male	43 (36.8)	40 (41.2)	3 (15.0)
Non-binary	1 (0.9)	1 (1.0)	0 (0.0)
Body mass index; kg/m^2^ mean (SD)	26.3 (5.4)	26.6 (5.6)	24.9 (4.2)
**Immunosuppressive therapy;** ***n*** **(%)**			
Rituximab Infusion (past six months)	38 (32.5)	38 (39.2)	0 (0.0)
Cyclophosphamide	7 (6.0)	6 (6.2)	1 (5.0)
Tocilizumab	6 (5.1)	0 (0.0)	6 (30.0)
Mepolizumab	4 (3.4)	4 (4.1)	0 (0.0)
Azathioprine	16 (13.7)	16 (16.5)	0 (0.0)
Methotrexate	25 (21.4)	21 (21.6)	4 (20.0)
Aprelimast	0 (0.0)	0 (0.0)	0 (0.0)
**Prednisolone;** ***n*** **(%)**			
>7.5 mg/day	11 (9.5)	8 (8.3)	3.0 (15.0)
≥5 and ≤ 7.5 mg/day	35 (30.2)	31 (32.3)	4.0 (20.0)
< 5 mg/day	33 (28.4)	25 (26.0)	8.0 (40.0)
Inhaled corticosteroids (alone or in combination)	4 (3.4)	4 (4.2)	0.0 (0.0)
Other	10 (8.6)	6 (8.3)	1 (10.0)
**Main provider of vasculitis care;** ***n*** **(%)**			
General practitioner	7 (6.5)	2 (2.2)	5 (26.3)
Nephrologist	13 (12.0)	13 (14.6)	0 (0.0)
Rheumatologist	87 (80.6)	73 (82.0)	14 (73.7)
**Main provider of other medical issues;** ***n*** **(%)**			
Dermatologist	1 (0.9)	1 (1.1)	0 (0.0)
General practitioner	77 (71.3)	63 (70.8)	14 (73.7)
Nephrologist	5 (4.6)	5 (5.6)	0 (0.0)
Neurologist	1 (0.9)	0 (0.0)	1 (5.3)
Rheumatologist	10 (9.3)	7 (7.0)	3 (15.8)
Other	14 (13.0)	13 (14.6)	1 (5.3)
**Current status;** ***n*** **(%)**			
Disease in remission	76 (76.0)	66 (79.5)	10 (58.8)
Active disease	24 (24.0)	17 (20.5)	7 (41.2)
**Disease progression;** ***n*** **(%)**			
Relapsing	43 (44.3)	35 (43.2)	8 (50.0)
Persistently active	10 (10.3)	7 (8.6)	3 (18.8)
In remission after initial therapy	44 (45.4)	39 (48.1)	5 (31.2)
**Overall health status;** ***n*** **(%)**			
Excellent	1 (1.0)	1 (1.1)	0 (0.0)
Very good	12 (11.4)	10 (11.5)	2 (11.1)
Good	43 (41.0)	36 (41.4)	7 (38.9)
Less good	39 (37.1)	30 (34.5)	9 (50.0)
Poor	10 (9.5)	10 (11.5)	0 (0.0)

Values shown are means ± SD or medians followed by the IQR, as indicated. The bold values indicate the numbers of the overall study population.

### Medical care during the COVID-19 pandemic

Most of the patients who participated in our study reported that their drug regimen included prednisolone at doses of < 5 mg/day (*n* = 33; 28.4%), 5–7.5 mg/day (*n* = 35/117; 30.2%), or >7.5 mg/day (*n* = 11/116; 9.5%). Rituximab was the most commonly used immunosuppressive drug (*n* = 38/117; 32.5%), followed by methotrexate (*n* = 25/117; 21.4%). Management of vasculitis was mainly provided by university hospitals (*n* = 53/106; 50.0%) and physicians in private practices (*n* = 37/106, 34.9%). Patient care was provided primarily by rheumatologists (*n* = 87/107; 81.3%) and to a lesser extent by nephrologists (*n* = 13/107; 12.1%). Most respondents identified general practitioners (GPs) as their main contact for all other medical concerns (*n* = 77/108; 71.3%). The patients reported that they needed to travel a median distance of 17 km (25%;75%quantile 10.0–40.0 km) to obtain care from the physician providing vasculitis care and 2.5 km (1.0–5.0 km) to receive care from their GPs (*n* = 108).

At the time of survey completion, 76% (*n* = 76/100) of the patients stated a stable disease status (remission). By contrast, 24% (*n* = 24/100) reported their disease status as active; 13 of these patients required recent adjustments to their therapeutic regimens to control the disease (*n* = 13/100, 13%). A relapsing disease was claimed by 43 of the patients (*n* = 43/97, 44.3%), while 44 patients (44/97; 45.4%) reported that they experienced only a single flare at the time of disease onset. The disease was classified as persistently active by 10 patients (*n* = 10/97, 10.3%).

### Changes in medical care due to the COVID-19 pandemic

Appointments were canceled due to COVID-19 in 13 patients (*n* = 13/103, 12.6%, 95% CI 7.3–20.0%); nine participants reported that their in-person appointments were replaced by digital services (e.g., remote consultations; *n* = 9/101, 8.9%, 95% CI 4.5–15.6%). Therapy was changed due to the pandemic in 18 respondents (*n* = 18/102, 17.6%; 95% CI 9.5–22.2%), including regimens that were shifted (*n* = 8/102; 7.8%), reduced (*n* = 6/102; 5.9%) or discontinued (*n* = 4/102; 3.9%). Therapy was changed in 16 of 84 patients (19.0%) with AAV and 2 of 18 patients with GCA (11.1%). In patient with a persistently active or relapsing disease (*n* = 53) therapy was changed due to COVID-19 in 9.4% compared to 27.3% (12/44) in patients with a stable disease. Table 2 displays the reported therapeutic modifications based on specific drug regimens received. Compared to other immunosuppressive therapies, therapy with Rituximab was more often postponed (17.2%) due to COVID-19.

**Table 2:** Modifications to therapeutic regimens due to COVID-19.

**Drug regimen**	**Yes, the start of therapy was postponed**	**Yes, the dosage or frequency was reduced**	**Yes, medication(s) was stopped**	**No modification**	**Total**
Azathioprine	0	1	0	14	**15**
Cyclophosphamide	1	0	0	0	**1**
Mepolizumab	1	0	0	2	**3**
Methotrexate	1	3	1	18	**23**
Rituximab	5	1	1	22	**29**
Tocilizumab	0	0	1	4	**5**
Others	0	0	0	10	**10**
**Total**	**8**	**5**	**3**	**60**	**76**

**Figure 1:**
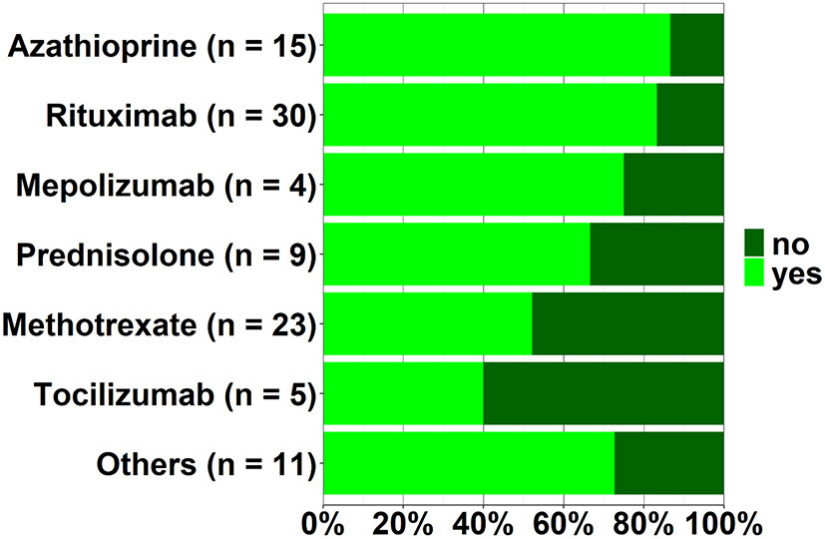
Respondents managed with specific drug regimens who reported concerns regarding increased susceptibility to COVID-19.

By the time the questionnaire was completed, 7.9% of the participants (*n* = 8/101, 95% CI 4.2–15.7%) had already had a COVID-19 infection. Interestingly, prevalence rates of COVID-19 among the general population in Germany were slightly higher (13%) compared to the participants included in this study^1^. Concerns regarding potential increased susceptibility to COVID-19 was stated by 70 participants (*n* = 70/104, 67.3%; 95% CI 57.9–75.8%); these concerns were most prevalent among patients undergoing treatment with azathioprine (13 of 15; 86.7%) or rituximab (24 of 29; 82.8%) (Figure 1). Concerns were not associated with patients age or gender. Patients with small-vessel vasculitis were more likely to report concerns (*n* = 61/85, 71.8%, 95% CI 61.6%; 80.5%) than patients with large-vessel vasculitis (*n* = 9/19, 47.4%, 95% CI 26.6%; 68.8%).

### Vaccination

Owning a vaccine record card was reported by 103 of the respondents (*n* = 103/104, 99.0%; 95% CI 95.6–99.9%). Vaccination status was checked most frequently by their GPs (*n* = 80/104; 76.9%; 95% CI 68.2–84.2%) or rheumatologists (*n* = 27/104; 26.0%; 95% CI 18.3–35.0%]). Vaccination status had not been checked by any physician in 15 patients (*n* = 15/104, 14.4%; 95% CI 8.7–22.1%). While eight participants (*n* = 8/100, 8.0%; 95% CI 3.7–14.0%) reported that they refused to undergo vaccination because of concerns regarding disease flares and/or side effects, the vast majority of patients (*n* = 92/104; 88.5%; 95% CI 81.3–93.5%) stated they had never refused vaccinations that were offered to them.

Patients were also asked about coverage for selected vaccinations (e.g., tetanus, pneumococcus, influenza, COVID-19; Table 3).

**Table 3:** Vaccination status.

**Vaccination**	**Eligible participants (n)**	**Eligible participants who were vaccinated** **n (%; 95% CI)**	**Vaccination coverage in the general population (%)**
**COVID-19**	103	100 (98.1; 93.9–99.6)	76.5^*^
Influenza (past year)^a^	78	65 (83.33; 73.9–90.3)	39.3^**^
Pneumococcus (past 5 years)^b^	78	49 (62.8; 51.8–72.9)	17.6^**^
Tetanus (past 10 years)	103	47^c^ (45.6; 36.2–55.3)	53.9^**^
Diphtheria (past 10 years)	103	23^c^ (22.3; 15.1–31.1)	52.7^**^

The reported frequencies are based on the number of persons eligible for the respective vaccination according to the recommendations of the German Society for Rheumatology and the German Standing Committee on Vaccination. The findings are compared to data obtained from the Association of Statutory Health Insurances from 2020/2021 (Kassenärztliche Vereinigungen, RKI Vaccination Surveillance);

a patients ≥60 years of age, currently managed with immunosuppressive therapy, or diagnosed with specific comorbidities (chronic cardiovascular, liver, renal or lung disease and/or diabetes);

b patients managed with immunosuppressive therapy;

c patients were asked if they have been vaccinated in the past 5 years;

* data obtained from the Robert-Koch institute,

** data obtained from Rieck et al. (17).

We used these data to compare their rates of vaccination with individuals in the general population. Compared with data from the Association of Statutory Health Insurances for the general population, the vaccination coverages were high in our cohort [e.g., 83% for influenza (*n* = 64/77, 95% CI 73.6–09.2) and 62.3% for pneumococcus (*n* = 58/77, 95% CI 51.2–72.5)] The highest vaccination coverage was reported for COVID-19 (*n* = 100/102, 98.0% 95% CI 93.9–99.6%). The coverage for COVID-19 vaccination was significantly higher compared to the general population in Germany (76.5%) at the time of survey completion on April 19, 2022, (Robert-Koch-Institute).

Seventeen patients (*n* = 17/94, 18%; 95% CI 10.9–25.7%) reported that they had not been informed by any of their physicians about COVID-19 vaccination; by contrast, 80 participants reported that they received information from their rheumatologists (*n* = 39/96; 40%; 95% CI 31.5–50.7%), GPs (*n* = 23/96; 24%; 95% CI 15.9–32.5%), or other physicians (*n* = 18/96; 19%; 95% CI 11.7–26.9%).

## Discussion

We report findings from a patient survey focused on the medical care provided to vasculitis patients during the COVID-19 pandemic. To the best of our knowledge, this is the first patient survey designed to assess (1) the changes in medical care provided to vasculitis patients in the COVID-19 pandemic and (2) vaccination acceptance in this patient cohort. While several published reports document the results of surveys that included patients with RMDs, the proportion of vasculitis patients was small and their responses were not analyzed separately; in other studies, only data from the first month of the pandemic were presented (18–22).

Participating patients in this study reported that prescheduled appointments for the disease vasculitis had been canceled due to COVID-19 (10.3%) or had been replaced by digital services (7.1%). The reported proportions are lower than those reported in a survey of rheumatologists throughout Europe that included patients with various RMD in which 82% stated that they had canceled patient appointments due to COVID-19 (23). Therefore, vasculitides might be considered as diseases that require closer monitoring compared to other RMDs.

At the beginning of the pandemic, patients and physicians faced substantial uncertainty due to the lack of data regarding the clinical course of COVID-19 in patients diagnosed with RMDs, most notably those managed with immunosuppressive therapy. The first findings to emerge suggested that rituximab therapy was associated with an increased risk of severe COVID-19 (8, 24, 25). Consequently, recommendations for a more stringent risk-benefit analysis were brought forward. However, recent studies have highlighted the extraordinary efficacy of rituximab for induction of remission and maintenance therapy in small vessel vasculitides (26–28). Rituximab was the most commonly used immunosuppressive drug in our patient cohort for patients diagnosed with AAV and remained the treatment of choice for 75.9% of the participants despite the pandemic.

For other immunosuppressive therapies, it was recommended to continue necessary treatments because ongoing disease activity was considered to pose a greater risk than COVID-19 infections (8). Only 17.6% (*n* = 18) of the respondents of our study reported changes in their therapeutic regimens (i.e., shifts, reductions, or discontinuations) due to COVID-19. Our results are consistent with previous reports that documented no major changes in the management of vasculitis patients during the COVID-19 pandemic (20, 29). This might be explained by the profound importance of current drug regimens used to prevent flare-ups in order to prevent irreversible damage or death in patients with uncontrolled vasculitis (30, 31).

Interestingly, the proportion of respondents who had already contracted and recovered from COVID-19 at the time the survey data was collected was slightly lower than that reported in the general population. These differences might be explained by the high proportion of participants who reported concerns regarding their potentially higher susceptibility to COVID-19. These individuals may have more stringently reduced their in-person social interactions and taken other safety precautions (2). Although the level of concern regarding their potentially higher susceptibility to COVID-19 was high in our cohort, a meta-analysis of data collected from studies that documented the prevalence of immunosuppression among patients with COVID-19 (*n* = 10,049) revealed that these patients were at no increased risk of contracting this infection (32). However, the meta-analysis did not explore the impact of different immunosuppressive therapies and diseases.

Overall, the study participants exhibited a positive attitude toward vaccinations. The majority (*n* = 92/104; 88.5%) stated they had never refused any vaccinations. The rate of COVID-19 vaccination was exceptionally high compared to previously reported studies focused on RMD patients (10–12) as well as in the general population in Germany^1^.

To interpret the COVID-19 vaccination rates in our cohort, patients were asked about other recommended vaccinations. We used these data to compare their rates of vaccination with individuals in the general population. Apart from the high COVID-19 vaccination rate in our study population, our findings also revealed a generally high rate of vaccination among members of our study, e.g. against influenza and pneumococcus (two- and three-fold higher, respectively). Compared to 2018 (i.e., before the COVID-19 pandemic), Harrison et al. (33) reported lower vaccination rates against influenza in RMDs patients. The increased acceptance of vaccination, in general, might be due to the increased information, public discussion and awareness of the need for vaccination during the COVID-19 pandemic as already observed by Starrostzik (34).

Among the strengths of our study, we note the comparatively large cohort size, patient recruitment based at multiple centers and collaboration with vasculitis patient advocacy groups to develop the survey. We do recognize the potential bias inherent in an online survey. In addition, there seems to be a bias toward patients with small-vessel vasculitis, as this disease is less common than large-vessel vasculitis in the general population (35, 36), but more prevalent in our cohort. This also explains the large proportion of patients reporting a therapy with rituximab, which is used as one standard therapy in ANCA-associated vasculitis. Since our survey was voluntary, more patients with concerns about COVID-19 infections may have participated in the survey.

## Conclusion

Participants of our self-reported survey experienced changes in medical care during the COVID-19 pandemic. Compared to the general population, acceptance of vaccinations, especially against COVID-19, was considerably high on our cohort.

## Data availability statement

The raw data supporting the conclusions of this article will be made available by the authors, without undue reservation.

## Ethics statement

The studies involving human participants were reviewed and approved by the Ethics Committee of the Medical Faculty of the University of Duesseldorf, Germany (2021-1405). The patients/participants provided their written informed consent to participate in this study.

## Author contributions

AK and TF performed the data analyses. AK and GC drafted the first version of the manuscript. All co-authors were involved in the critical interpretation of the results, discussed the findings together, critically reviewed the manuscript and approved its final version. All authors designed the study and contributed to data collection.

## Footnotes

^1^https://experience.arcgis.com/unsupported-browser/index.html (accessed November 11, 2022).
