# Supplementary material for: COVID-19 pandemic impairs medical care of vasculitis patients in Germany: Results of a national patient survey
Source: Front Med (Lausanne). 2023 Jan 9;9:1103694. doi: 10.3389/fmed.2022.1103694 (PMC9868561; doi:10.3389/fmed.2022.1103694)
Supplement: Supplementary file 1 [file Data_Sheet_1.PDF]

Patients characteristics

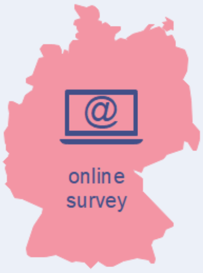

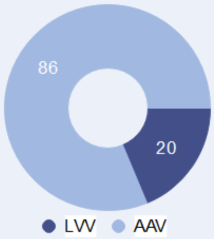

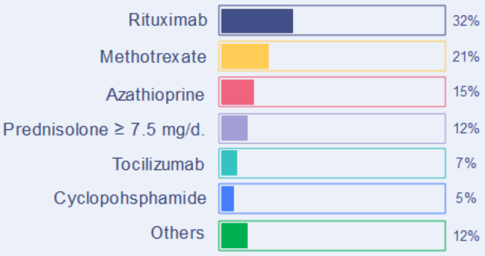

76%
in remission

10%
permanently active disease

"Due to COVID-19 i experienced ...

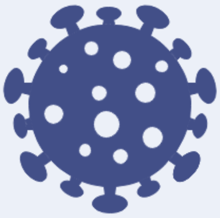

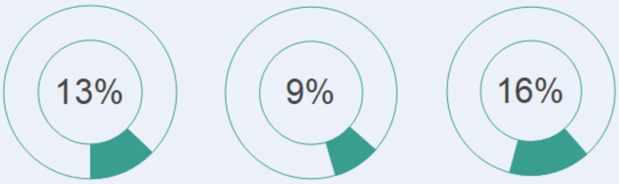

... cancelation of prescheduled appointments."

... visits replaced by digital services."

... changes in therapy."

"I'm vaccinated against ...

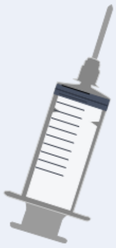

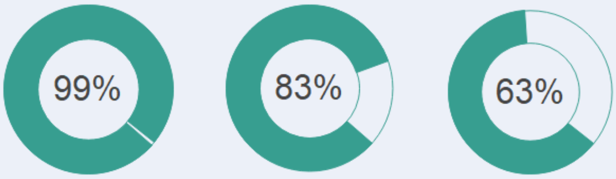

COVID-19

Influenza

Pneumococcus
